# Implementing Federal Immigration Medical Examinations into a United States Student-Run Free Clinic

**DOI:** 10.1007/s10900-025-01488-0

**Published:** 2025-06-09

**Authors:** Julio Siliezar, Paola Rodriguez, Yakelin Arroyo-Velazquez, Kimberly Aguirre Siliezar, Melanie Venegas, Carlos Melchor, Micah Stierli, Mark Diaz, Brenden Tu, Michael Wilkes

**Affiliations:** 1 University of California Davis School of Medicine, Sacramento, CA USA; 2 University of California Davis School of Veterinary Medicine, Davis, CA USA; 3 Department of Biological Sciences, California State University Chico, Chico, CA USA; 4 Department of Family Medicine, University of California Davis, Sacramento, CA USA; 5 Department of Internal Medicine, University of California Davis, Sacramento, CA USA

**Keywords:** Refugee, Free clinic, Medical education, Vaccination, Communicable diseases, Access to care

## Abstract

The Immigration Medical Examination (IME) is a mandatory step in obtaining U.S. permanent residency, yet its high cost and limited accessibility pose significant barriers for low-income immigrants. Addressing these challenges is critical to ensuring equitable healthcare access for underserved populations. To present the benefits and process of integrating IME services into a student-run free clinic serving low-income immigrant populations. A review of IME requirements, cost barriers, and existing student-run clinic models informed the development of an IME program at the Knights Landing One Health Center (KLOHC). The implementation process involved four key steps: assessing community needs, securing qualified personnel, establishing clinic logistics, and implementing a structured training model for student volunteers. A retrospective chart review of patients receiving IME services from May 2022 to May 2024 was conducted to analyze patient demographics, service utilization, and health screening outcomes. KLOHC provided IME services to 204 patients, with 177 adult records analyzed. The majority were Hispanic/Latino (84%) and Spanish-speaking (79%), highlighting the importance of culturally and linguistically competent care. Patient volume steadily increased, with a consistent waitlist of 14–20 individuals per session. Cost reduction was significant, with services provided at no cost for selected cities already served by KLOHC. These cities include Knights Landing, Davis, Winters, Woodland (Yolo County), Yuba City (Sutter County), and Sacramento (Sacramento County). A $250 was charged to non-target community patients, compared to the private sector’s $400–$900 range. IME health screenings identified cases of tuberculosis and sexually transmitted infections, enabling timely treatment. The structured student leadership model ensured continuity, training, and sustainability. Integrating IME services into student-run clinics is a feasible and effective strategy to expand access to essential health evaluations for immigrant populations. Funds collected from non-target communities were used to pay for vaccines, x-rays, and transportation for target community members which eased the financial burden of the already expensive immigration process. KLOHC’s model demonstrates a cost-effective service for patients, improves healthcare accessibility, and provides hands-on training for future healthcare professionals. This initiative serves as a replicable framework for other student-run clinics nationwide, addressing a critical gap in immigrant healthcare and promoting a more equitable healthcare system.

## Introduction

The process to obtain citizenship in the United States is often lengthy and complex, posing challenges for immigrants who play a key role in addressing the nation’s historically low population growth [1]. U.S. Census data shows that the immigrant population increased from 376,000 in 2020-21 to 1.14 million in 2022-23 [2, 3]. Central to this path to permanent residency or a “green card” is the Immigration Medical Examination (IME), known as the “Report of Immigration Medical Examination and Vaccination Record United States Citizenship and Immigration Services (USCIS form I-693)”, a mandatory health evaluation that ensures public health and safety [4, 5]. These assessments, performed by USCIS-authorized civil surgeons, include physical and mental health evaluations, drug and mental health screenings, immunization reviews, and infectious disease testing [4, 6, 7]. Obtaining a green card is the first step in a multi-year immigration process that culminates in naturalization for those seeking full citizenship [8].

With the immigrant population surging, including 1,172,910 individuals granted permanent U.S. residence and 878,500 granted U.S. Citizenship in 2023—the highest naturalization totals since 2008—there is considerable strain on the U.S. immigration system [2, 9, 10]. Nearly 100,000 individuals—approximately 9% of those granted permanent residency in 2023—were refugees or asylees, with the majority residing in Texas and California [11, 12]. Additionally, one-fifth of those granted permanent residence status in 2023 live in California, all requiring IMEs [9, 10, 13, 14]. These IMEs vary in cost across the U.S. but in Sacramento County they are between $400–$900 per exam. These services are often inaccessible to low-wage immigrant workers, as they are not covered by health insurance [5, 15]. Recognizing this pressing need, the Knights Landing One Health Center (KLOHC)—a UC Davis School of Medicine affiliated student-run free clinic established in 2011 to serve predominantly Latino farmworkers in rural Northern California—began offering IMES in 2022. These services are overseen by a USCIS-authorized civil surgeon and supported by UC Davis undergraduate and medical students, ensuring linguistically competent, culturally sensitive care for underserved and immigrant populations while fostering student education in rural health [16–18].

Student-run clinics have proved to be successful avenues for bringing quality primary care, psychiatry, general surgery and plastic surgery services to vulnerable populations [19–23]. The addition of IME services at KLOHC follows on these ideas and seeks to not only ease the financial and logistical burdens on immigrants but also serves as a scalable model for other student-run clinics. This work addresses the national need for expanding IME services and highlights the benefits of replicating the KLOHC model. Key considerations such as service accessibility, cost-effectiveness for patients, and the clinic’s sustainability through medical student volunteers are analyzed to support broader implementation of this vital service.

## Methods

This framework was developed using the IME service at KLOHC as a foundational model. Service logistics were designed to promote the clinic within the local Knights Landing community and later expanded to serve neighboring cities such as Yuba City, Woodland, and Davis. Outreach efforts also targeted local migrant camps. The framework outlined below is organized into four distinct steps, followed by a chart review of patients who received immigration medical examination services at the clinic.

1. Assessing geographical need and target service region.

The first step in implementing an immigration medical examination service is assessing the community’s need, which informs staffing, logistics, and workflow. Partnering with community organizations that serve underserved populations helps identify areas needing medical services, builds a local presence, and creates a network of resources. A clear mission statement outlining the clinic’s goals and impact helps gain public support. Once the need is established and support is secured, a formal agreement with the partnering university can be made.

Target communities were identified based on the service area were most KLOHC patients come from. These included the cities of Knights Landing, Davis, Winters, Woodland (Yolo County); Yuba City (Sutter County) as well as the city of Sacramento (Sacramento County) and other UC Davis–affiliated student-run clinics in Sacramento. The IME clinic quickly gained visibility, with the waitlist expanding after just the second month of service in July 2022. Community college students from Sacramento and Yolo Counties, along with students from the University of California, Davis, and Sacramento State University, were also considered part of the target population. This inclusion was due to the fact that approximately two-thirds of the student population in the area typically have limited income and belong to sizable immigrant communities [24, 25].

2. Obtaining qualified medical personnel.

Securing dedicated medical personnel is key to ensuring high-quality care. The IME at KLOHC addressed this by opening clinical volunteer positions for the University of California, Davis pre-medical and medical students. Preceptors from UC Davis School of Medicine and local physicians were invited to volunteer so qualified and multilingual, local volunteers were recruited to assist with interpretation services.

After assembling a motivated team, a qualified physician was appointed to oversee clinic organization and sustainability. For IME services, a licensed physician authorized by USCIS as a civil surgeon was required, as civil surgeons are responsible for conducting immigration medical examinations according to USCIS technical instructions [26]. The process of becoming a designated civil surgeon through USCIS can take up to two years and involves meeting specific criteria such as being board certified in any medical specialty and having practiced 5 years (post-residency) as a fully licensed physician in the U.S [27].

3. Logistics and clinic expenses.

Once the clinic had a civil surgeon and volunteer staff, physicians and students established operational days, staffing levels, and community outreach. Promotoras, local community volunteers, played a key role in raising awareness and building trust in KLOHC. The IME clinic operates monthly based on volunteer and physician availability, usually on weekends, and serves approximately 15 patients per day.

IME services are offered free of charge to patients from the communities identified in the “Assessing Geographic Need and Target Service Area” section. Patients from outside the target region pay a reduced fee of $250, which covers a physical examination, laboratory testing (for tuberculosis, chlamydia, gonorrhea, and syphilis), blood antibody testing, and completion of USCIS Form I-693. This fee is substantially lower than those charged by private civil surgeons, which typically range from $600 to $900 per person per exam. This estimate was obtained by contacting multiple private civil surgeons in Yolo and Sacramento counties. These private fees often exclude vaccines or laboratory testing. Revenue from non-resident fees supports clinic supplies, vaccine reimbursements, and patient transportation. Vaccines, which often cost more than $100 each, represented the clinic’s most significant expense, as most uninsured patients required multiple immunizations [28–30].

Additional funding was acquired from the Physicians for a Healthy California MedStudentsServe grant, which was used to purchase essential office and medical supplies such as printers, toner, stamps, and supply carts, stethoscopes, blood pressure machines and reflex hammers [31]. In total an initial budget of approximately $3000 was secured to establish the clinic.

4. Implementing structural roles and responsibilities.

To establish a sustainable and permanent clinical examination system, KLOHC formed a committee of medical students from all four years and undergraduate volunteers. Undergraduate volunteers provide clinic staffing and are trained in immigration eligibility criteria necessary to complete Form I-693. Medical students took on increasing responsibilities based on their year:

- First-years: conduct patient examinations, supervise and train undergraduate volunteers.
- Second-years: manage clinic flow, lead patient outreach, and train first-years.
- Third-years: provide training and classes on Form I-693 and its associated screenings for first-years, second-years, and undergraduates.
- Fourth-years: oversee workflow improvements, grant writing, support third-years, and address issues like patient satisfaction and clinic expansion to surrounding communities.

This organized system ensures high-quality care and instruction while promoting sustainability through student engagement. Medical students develop diagnostic reasoning skills, and undergraduates gain valuable clinical experience in a structured, hands-on environment [32–34].

### Sample Analysis

Patient data for this study was collected during initial IME visits at KLOHC and entered into the clinic’s electronic medical record (EMR) system. The analysis reviewed patient records from May 2022 to May 2024, excluding external or additional data sources. Patients located the clinic through the online USCIS “Find a Civil Surgeon” tool or through community and word-of-mouth referrals in surrounding cities of Knights Landing, Yuba City, Woodland, or Davis.

As part of the IME, adult patients were screened for communicable diseases categorized by the Centers for Disease Control and Prevention (CDC) as Class A or B conditions. Class A conditions include untreated communicable diseases (e.g., tuberculosis, HIV, syphilis, gonorrhea, Hansen’s disease), rendering a patient inadmissible. Class B conditions include treated or controlled diseases (e.g., latent tuberculosis infection, chronic conditions) and do not bar admission. Screening methods included QuantiFERON-TB Gold+ (tuberculosis), syphilis RPR, and urine tests for chlamydia/gonorrhea, all of which were performed by the UC Davis Health Lab. Positive results for tuberculosis led to chest X-rays and referral to county health departments for treatment. Positive syphilis, chlamydia, or gonorrhea results were treated at KLOHC [35–38].

Vaccination requirements followed CDC technical instructions [39]. Patients needed proof of vaccination or laboratory immunity (e.g. titer levels) for listed vaccines. Missing vaccines in a series required the first dose before completing Form I-693, but series completion was not mandatory to complete the immigration medical requirements [26].

## Results

A total of 204 patients were seen between May 2022 and May 2024 at the Knights Landing One Health Center. Twenty-seven charts belonging to minors (age 18 years or younger) were excluded from review, leaving a total of 177 adult patients that were included for final analysis. Demographic information for the 177 adult patients is shown in Table 1. The majority of patients were female (108, 61%), Hispanic/Latino (149, 84%), and 30 years old or older (133, 75%). Ten different languages were represented in our patient population, with Spanish being the most spoken language (140, 79%). Most patients (131, 74%) opted to use an interpreter for their exam. Patients had a variety of immigration statuses, with the top three most common statuses being ‘Pending Adjustment of Status under Sect. 245 of the Act’ (119, 67%), ‘DACA’ (13, 7%), and ‘Asylee’ (11, 6%).

**Table 1:** Demographic information for 177 adult patients seen at the KLOHC IME clinic from May 2022-May 2024

	Count (*n* = 177)	Percent
*Gender*		
Female	108	61%
Male	69	39%
*Age (years)*		
18–29	44	25%
30–49	71	40%
50–64	57	32%
65+	5	3%
*Ethnicity*		
Hispanic/Latino	149	84%
Non-Hispanic/Latino	28	16%
*Immigration Status*		
Asylee	11	6%
Asylum Application Pending	9	5%
DACA	13	7%
Deferred action	2	1%
Family Unity Program (Sect. 301 of the Immigration Act of 1990)	1	1%
Foreign Fiancé(e)	1	1%
None	1	1%
Paroled refugee	1	1%
Pending Adjustment of Status under Sect. 245 of the Act	119	67%
Public Interest parolee	9	5%
Temporary Protected Status	1	1%
U-1 nonimmigrant	4	2%
U-2, U-3, U-4, or U-5 nonimmigrant	1	1%
VAWA self-petitioners with an approved Form I-360	4	2%
*Primary Language*		
Chinese	1	1%
Dari	7	4%
English	21	12%
French	1	1%
Indonesian	1	1%
Portuguese	1	1%
Punjabi	3	2%
Russian	1	1%
Spanish	140	79%
Tamil	1	1%
*Interpreter Used*		
Yes	131	74%
No	46	26%

**Fig. 1:**
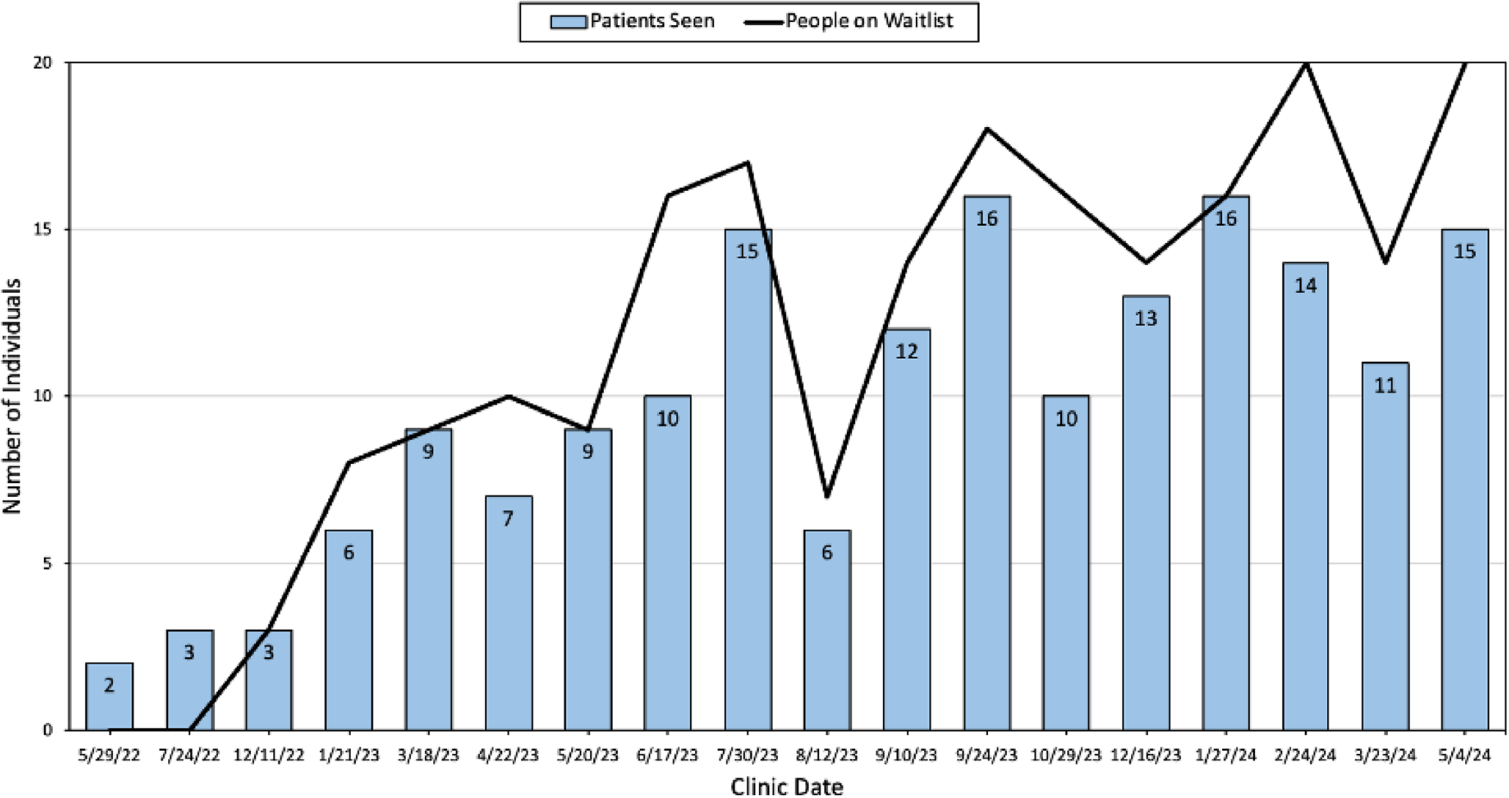
Number of patients seen for immigration medical examinations at the Knights Landing One Health Center with corresponding people on the clinic waitlist, from May 2022 to May 2024. Months when clinic examination services were not in effect were excluded from the chart

From May 2022 to July 2023, there was a steady increase in the number of patients seen at the IME clinic and a steep increase in the number of individuals on the clinic waitlist (Fig. 1). After a steep drop in August 2023, the number of patients seen at the IME clinic varies but in general stabilizes around 10 to 16 patients per clinic date. Similarly, after August 2023, the number of individuals on the waitlist fluctuates greatly between 14 and 20 individuals per clinic date.

**Fig. 2:**
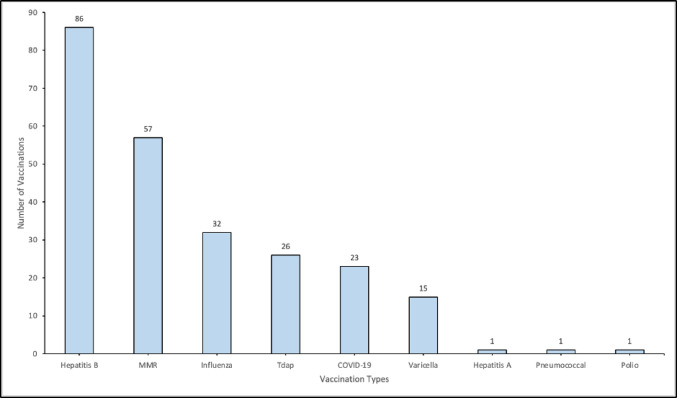
Most common vaccinations that were missing/incomplete for patients coming to the KLOHC IME clinic, from May 2022 to May 2024

Communicable diseases are a central theme of form I-693. The results of the screening tests for tuberculosis, syphilis, chlamydia, and gonorrhea are listed in Table 2. For the aforementioned diseases, screening tests were performed on 176 instead of the total 177 individuals, due to one patient having completed all laboratory screening tests prior to their visit. Twenty-nine patients (16%) tested positive for tuberculosis on QFT and required subsequent chest X-rays. All 29 were negative for pulmonary TB, diagnosed with LTBI, and referred to their respective county’s department of public health. Along with tests for communicable diseases, vaccinations are the second most important screening criteria for form I-693. The top three most commonly missed vaccinations were the Hepatitis B, (86, 49%), MMR (57, 32%), and Influenza (32, 18%) vaccines (Fig. 2).

**Table 2:** Test results summary (count and prevalence) for communicable diseases screened for as part of the KLOHC immigration medical exam

	Positive	Negative
Tuberculosis	29 (16%)	147 (84%)
Syphilis	5 (3%)	171 (97%)
Chlamydia	2 (1%)	174 (99%)
Gonorrhea	0 (0%)	176 (100%)
Hansen’s Disease	0 (0%)	177 (100%)

**Fig. 3:**
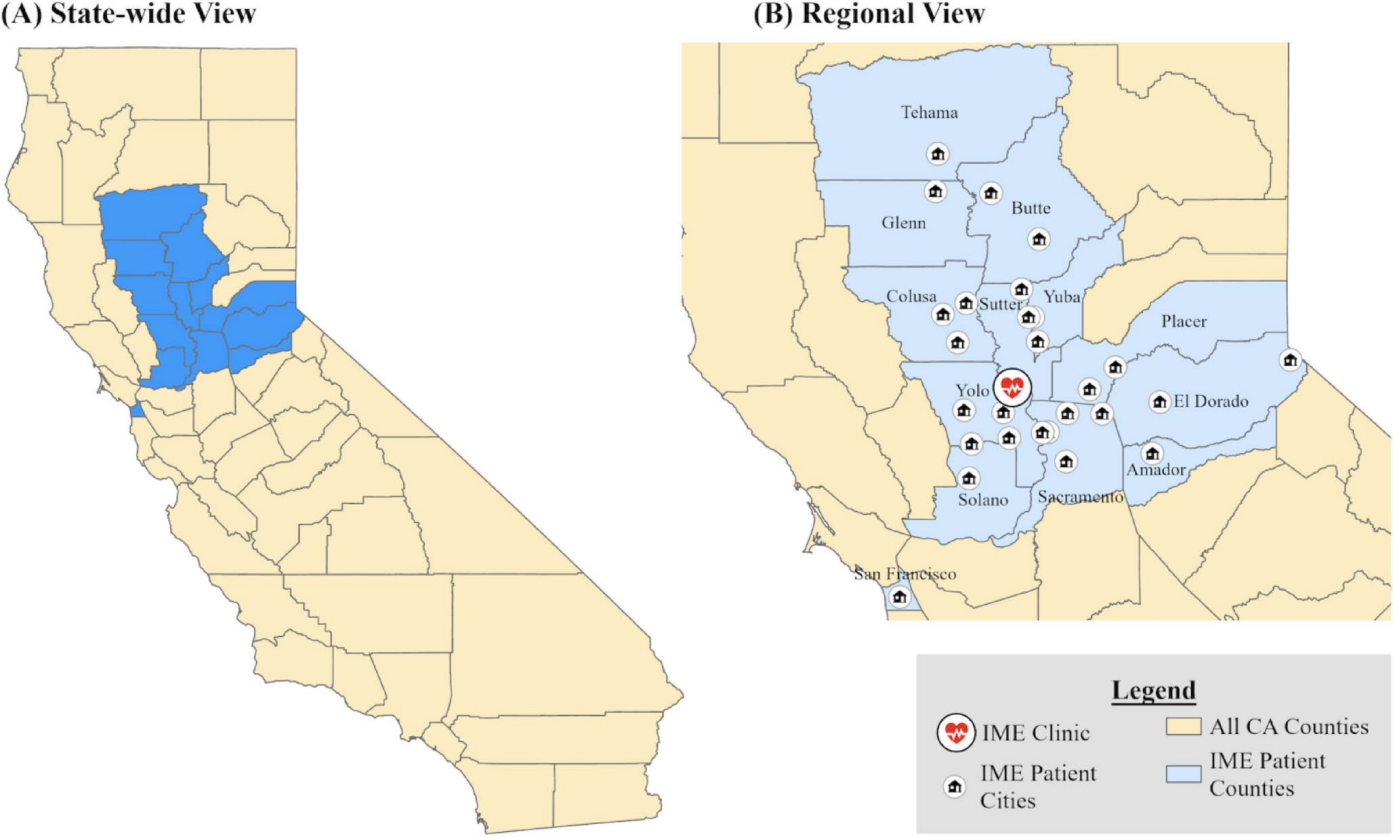
California counties and cities of residence of patients attending the KLOHC IME clinic, from May 2022 to May 2024. (A) State-wide View shows counties across the entire state, while (B) Regional View shows counties and cities in more detail, as well as the location of the IME clinic

Initial clinic outreach focused on the local populations of Sacramento, Winters, Davis, Woodland, Yuba City, and Knight’s Landing. The need for the exam quickly grew as demonstrated in Fig. 3, and patients started reaching out from various cities across northern CA. Most came from surrounding cities close to Knights Landing, while others came as far away as San Francisco (92 miles away) and South Lake Tahoe (129 miles away). Figure 3 presents a map of the 29 California cities where most of our patients came from. Not depicted in the figure are two patients coming from outside of California (one from Carson City, Nevada and another from Cincinnati, Ohio).

## Discussion

The primary objective of this paper is to demonstrate how student-run clinics can utilize available resources to implement an efficient IME service for immigrant communities.

A key strength of this model is the synergy between clinic volunteers and local immigrant communities. Through direct involvement in the IME process, students not only help immigrants achieve an essential step toward U.S. citizenship but also gain valuable clinical experience with diseases uncommon in the general U.S. population, such as tuberculosis and Hansen’s disease. This early patient interaction, especially during pre-clinical years, offers students a more hands-on and immersive learning experience.

Moreover, this community-centered approach enhances trust-building and communication between healthcare providers and immigrant populations, addressing a critical barrier to care in underserved communities.^18^ Medical and undergraduate students involved in the clinic can additionally develop a deeper understanding of the challenges faced by these populations, which in turn strengthens the physician-patient relationship, reduces personal biases, and promotes culturally competent care.

Beyond clinical care, the implementation of this model promotes community-based participatory research (CBPR), a method crucial to addressing health inequities in underserved populations.^19^ By fostering partnerships with local organizations, encouraging community input, and sharing resources, this approach allows the clinic to continuously evolve and respond to the changing needs of the population it serves. Such a model holds promise for improving health outcomes in low-income immigrant communities through creating a sustainable and culturally sensitive framework for care.

The first two clinics, held in May 2022 and July 2022, had few patients. To address this, outreach initiatives were implemented, and a waitlist for the clinic was created shortly afterward to better accommodate patients visiting KLOHC. This shows the successful involvement of the community in outreach initiatives and the importance of CBPR in implementing projects such as this around the U.S. Figure 3 illustrates the growing demand for the IME service at the Knights Landing One Health Center, as evidenced by its annually expanding patient base. The affordability of the IME implementation process makes it a viable and replicable business model for other facilities. Moreover, the framework’s flexibility allows it to be adapted to meet the unique needs of different communities.

Communicable diseases are a central focus of USCIS Form I-693, with tuberculosis (TB) recognized as the world’s leading infectious killer [40]. According to the World Health Organization (WHO), approximately 10 million people contract TB annually, and despite being preventable and treatable, 1.5 million die from the disease each year [40, 41]. Furthermore, an estimated 10% of individuals with latent tuberculosis infection (LTBI) will develop active TB later in life [42]. The California Department of Public Health (CDPH) reports a 24% increase in active TB cases since 2020, with 85% resulting from the progression of LTBI to active disease [43]. Notably, individuals born outside the U.S. have a TB rate 13 times higher than U.S.-born individuals, underscoring the importance of identifying and treating LTBI to protect public health [43, 44]. In Table 2, we observed that 29 patients (16%) were diagnosed with LTBI and referred for treatment in their respective counties. Table 2 also shows that the prevalence of TB in our population was higher than that of syphilis (3%) and chlamydia (1%), the other two communicable diseases screened for, making TB screening the most critical during the IME. Clinics, such as KLOHC, that screen immigrants for TB and LTBI provide a critical public health service, acting as a first line of defense in identifying and addressing this burden of disease while highlighting the importance of building close ties to the communities we serve [44].

Through adopting a system tailored to the unique demographic needs of underserved populations, clinics similar to the KLOHC could bridge gaps in healthcare access and foster deeper connections between medical professionals and communities.

## Limitations

Data collected for this work is limited by the unique setting and specific service offerings of KLOHC. KLOHC is situated in a rural community of approximately 1000 people making community engagement easier which may not be directly comparable to other geographical regions or clinics. California is home to 23% of the 46 million immigrants residing in the United States. The relatively small sample size of patients receiving IME services at KLOHC does not reflect the overall immigrant population size in Northern California, where immigration is particularly high. This may not be representative of states with smaller immigrant populations or different healthcare needs. Our patient population was predominantly Latino, originating mostly from Mexico, which might not reflect other immigrant communities across the U.S.

Furthermore, implementing this framework in other clinics, especially in states with fewer resources or smaller immigrant populations, presents challenges such as funding, availability of civil surgeons and differing academic responsibilities for students in their early years of study. As such, further research is required to explore the best practices for adapting this model into student-run clinics at other medical schools across the U.S., taking into account regional and institutional variations.

## Conclusion

The aim of this work is to provide a detailed description and framework that could aid in enabling other student run eligible clinics around the U.S. to implement their own IME service. Integrating this service has the potential to remedy the lack of free/discounted services offering completion of the USCIS form I-693 to recent refugees, asylees, or victims of human trafficking. Most student-run clinics are dedicated to serving specific demographics, with volunteers passionately working to build trust and respect within these communities through intentional actions and outreach. This makes the work of establishing, staffing and continuing an IME clinic much easier. Additionally, the possibility of establishing this service around the U.S., specifically in states with sizable immigrant communities such as California, Texas, Florida, and New York, provides an avenue to help the already strained U.S. immigration system while at the same time providing motivated medical students the opportunity to directly impact the wellbeing of the communities they serve.

As immigrants become permanent residents in the U.S. they are able to integrate much more successfully into society, access essential services such as health insurance, and advance economically. All these benefits have the possibility of also contributing to a healthier society by identifying communicable and chronic disease processes earlier in their development and providing interventions sooner than would have been possible. Overall, the integration of this IME system contributes to the development of a healthier society and assists in reducing persistent health disparities in our healthcare system.
